# Barriers to physical activity in patients with pulmonary hypertension

**DOI:** 10.1177/2045894019847895

**Published:** 2019-05-17

**Authors:** Thomas M. Cascino, Vallerie V. McLaughlin, Caroline R. Richardson, Nilofar Behbahani-Nejad, Victor M. Moles, Scott H. Visovatti, Elizabeth A. Jackson

**Affiliations:** 1 Division of Cardiovascular Medicine, University of Michigan, Ann Arbor, MI, USA; 2 Department of Family Medicine, University of Michigan, Ann Arbor, MI, USA; 3 Oakland University William Beaumont School of Medicine, Auburn Hills, MI, USA; 4 Division of Cardiovascular Medicine, University of Alabama at Birmingham, Birmingham, AL, USA

**Keywords:** Exercise capacity, barriers, motivation

## Abstract

Patients with pulmonary arterial hypertension (PAH) and chronic thromboembolic pulmonary hypertension (CTEPH) have low levels of physical activity (PA). Increased PA has health benefits including improved quality of life. This study aimed to identify patient-perceived barriers to PA that correlate with objectively measured PA in this population. We performed a cross-sectional survey of 40 patients with PAH and CTEPH. Participants rated how often 15 barriers interfere with being physically active on a 5-point Likert Scale. The primary outcome measure was PA quantified using the Fitbit Zip activity tracker for two weeks. The primary independent variables were the 15 barriers and a summary score (total average barriers). Separate multivariable linear regressions were performed to assess the association between the 15 barriers and the summary score and PA adjusting for age, sex, and PAH etiology. Of the participants, 85% (34/40) had valid step counts and were included. Of these 34, 85% (n = 29) were female and 91% (n = 31) had PAH. The median (interquartile range [IQR]) number of daily steps was 3913 (2309–6313). The barriers endorsed most strongly were lack of self-discipline, lack of energy, and lack of interest. In the multivariable analysis, a 1-unit increase in perceived lack of interest, lack of enjoyment, and lack of skills was associated with a significant decrease in step counts of -1414 steps (95% confidence interval [CI] = (−2580 – −248), −1458 steps (−2404 – −511), and −1533 steps (−2910 – −156), respectively. Counseling and interventions aimed at increasing PA in patients with PAH should address interest, enjoyment, and skill development.

Patients with pulmonary arterial hypertension (PAH) and chronic thromboembolic pulmonary hypertension (CTEPH) have low levels of physical activity (PA) and low health-related quality of life (HRQOL)^1^ with increased sedentary time and reduced PA compared to controls.^1–3^ The overall benefits of PA in the management of a diverse range of cardiovascular diseases is well established.^4^ Lower PA has recently been shown to be associated with worse patient-reported symptoms in patients with PAH.^1,5^ This has led to an interest in treatments aimed at increasing the daily PA of patients with pulmonary hypertension (PH) to improve functional status and HRQOL.^6^

Current PH guidelines recommend encouraging PA while avoiding distressing symptoms or excessive exertion.^7,8^ Interventions aimed at increasing PA have largely focused on the pharmacological management of PH or structured exercise therapy.^9^ While improved use of these therapies has the potential to increase PA, there are likely additional personal and environmental barriers that exist, similar to the general adult population.^10^ An improved understanding of perceived barriers for patients with PH becomes critical in developing interventions aimed at increasing PA.

Accordingly, the purpose of the study was to identify patient-perceived barriers to PA in patients with PH. We hypothesized that patient-perceived barriers to PA would correlate with objectively measured PA.

## Methods

### Study design

We performed a cross-sectional study of patients with PAH and CTEPH. Participants were recruited from the University of Michigan Medical Center pulmonary hypertension clinic between June 2016 and November 2016 as part of a more extended prospective study designed to explore long-term associations between PA and quality of life. The study was approved by the University of Michigan Institutional Review Board and written informed consent was obtained from all patients (HUM00110649).

### Participants

We enrolled patients aged ≥18 years who had a diagnosis of PAH or CTEPH by right heart catheterization according to World Health Organization (WHO) Criteria,^7^ WHO functional class (FC) I–III symptoms,^11^ and a stable PAH-specific medication regimen for at least three months. Participants were excluded if they had a life expectancy <1 year, co-morbidities which limit physical activity to a severe degree (e.g. wheelchair-bound), current uncontrolled psychiatric disease, active substance abuse, concurrent enrollment in pulmonary rehabilitation, or current pregnancy.

### Measures

Basic demographic characteristics including age, sex, co-morbid conditions, medications, WHO FC,^11^ and 6-min walk distance (6MWD) at the time of enrollment were ascertained from review of the medical record. All participants completed a 6-min walk test in a standardized manner by trained personal consistent with the American Thoracic Society guidelines.^12^

### Primary outcome

Average daily PA was measured using the Fitbit Zip™ accelerometer over the course of two weeks. The Fitbit Zip™ is a commercially available device that can be attached to the participant’s waistband, belt, shirt, or wrist. The device has been shown to reliably and validly measure activity in free-living conditions.^13^ Participants were instructed to continue their usual level of activity while wearing the pedometer during waking hours. Participants were specifically not given step count goals or PA outputs. Reactivity, the change in a person’s PA after beginning to use a pedometer, was assessed and found not to be present.^14^ Thus, the two-week PA monitoring period began on the day of enrollment. Daily and weekly step counts were included in the analysis if the participant walked ≥200 steps on at least 4/7 days during the week. The lower limit of 200 steps was selected after inspection of the raw data and was 2 SD below the mean daily step count for the participants. For descriptive purposes, the participants were classified into “low” if below, or “high” if above, the median average daily step count for the cohort.

### Assessment of barriers to physical activity

Perceived barriers to exercise and PA were assessed with a 15-item questionnaire of established barriers described by Salis, et al. in the general population^15^ and rated on a 5-point Likert scale in the range of “1 = never” to “5 = very often.” Participants were asked how often each barrier interfered with, or prevented them from, exercising or being physically active. The internal consistency was high with a Cronbach’s alpha of 0.92. In order to assess overall barriers to PA, the total barriers score was determined from the average of the 15 barriers.

### Statistical analysis

Participant demographic characteristics, 6MWD, PA level, and barriers were collected as frequencies for categorical variables and means for continuous variables. Baseline characteristics and univariable analysis of patient-perceived barriers were analyzed according to PA level (based on median step count) using Wilcoxon rank-sum test for continuous variables and Pearson’s chi-squared test for categorical variables. Step counts between weeks 1 and 2 compared with a paired t-test. The frequency of a barrier reported as “often” or “very often” was determined.

The association between patient perceived barriers and PA as a continuous variable was evaluated with unadjusted and adjusted linear regression. For all estimated models, the dependent variable was PA, quantified as the average daily step count. Separate regressions were performed with each patient-perceived barrier as the primary independent variable. A final model was done using the total average barriers score as the primary dependent variable. All models were adjusted for age, sex, and etiology of PH in the multivariable analysis. Etiology of PH was adjusted for as scleroderma-associated PAH has previously been associated with lower levels of PA.^5^ Regression coefficients representing the change in average daily step counts per a 1-unit increase in barriers (e.g. going from “never” to “rarely”) were generated. Using the final linear regression models, estimations of the marginal mean effect of perceived barriers on PA, adjusting for age, sex, and etiology of PH, were estimated using the Stata (v15) margins command. After completion of the primary analysis, an exploratory analysis was performed with sub-maximal exercise testing (6MWD) as the dependent variable given the prior described association between average daily step counts and 6MWD.^1–3,5^ A *P* value <0.5 was used to determine statistical significance. Analyses were conducted using STATA 15 (College Station, TX, USA).

## Results

In total, 40 participants were enrolled. Of the 40 participants, 34 (85%) had valid step counts and were included in the analysis. Participant demographic and clinical characteristics are listed in Table 1. The majority of the participants had PAH (n = 31, 91%) that was either idiopathic (n = 13, 38%) or associated with connective tissue disease (n = 15, 44%) and were female (n = 29, 85%) with a mean age ( ± SD) of 61 (± 12). The median step count for the cohort was 3913 (interquartile range [IQR] = 2309–6313) steps. Step counts in weeks 1 and 2 were similar (mean difference = 220 steps, 95% confidence interval [CI] = −346–788, *P* value >0.20). When looking at the characteristics of the cohort, the average age, race/ethnicity, and vital signs were similar between participants grouped by activity level (Table 1). Notable differences between the groups included lower BMI and better 6MWD in the high activity group and more connective-tissue disease associated PAH, worse FC, and increased diuretic use in the low activity group.

**Table 1.** Baseline characteristics grouped by PA level.

Characteristics	Total (n = 34)	High activity (n = 17)	Low activity (n = 17)	*P* value
Age (years) (mean (SD)	61 (12)	59 (13)	63 (11)	0.32
Female	29 (85)	13 (76)	16 (94)	0.15
Race				
Black	4 (12)	2 (12)	2 (12)	1.00
White	27 (79)	14 (82)	13 (76)	
Not reported	3 (9)	1 (6)	2 (12)	
BMI (kg/m^2^) (mean (SD)	28 (5)	25 (3)	31 (6)	0.001
SBP (mmHg) (mean (SD)	123 (19)	119 (18)	127 (20)	0.23
DBP (mmHg) (mean (SD)	64 (9)	65 (9)	64 (9)	0.98
HR (BPM) (mean (SD)	78 (16)	76 (16)	81 (16)	0.34
PH type				
PAH				0.013
Idiopathic	13 (38)	9 (53)	4 (24)	
Heritable	1 (3)	1 (6)	0 (0)	
CTD	15 (44)	3 (18)	12 (71)	
Portal hypertension	1 (3)	1 (6)	0 (0)	
CHD	1 (3)	1 (6)	0 (0)	
CTEPH	3 (9)	2 (12)	1 (6)	
WHO FC				0.035
Class I	5 (15)	4 (24)	1 (6)	
Class II	21 (62)	12 (71)	9 (53)	
Class III	8 (24)	1 (6)	7 (41)	
Steps per day (mean (SD))	4650 (3246)	6962 (3052)	2337 (1027)	<0.001
6MWD (m) (mean (SD))	465 (115)	548 (74)	378 (79)	<0.001
Medications				
Prostacyclins	15 (44)	8 (47)	7 (41)	1.00
ERA	18 (53)	9 (53)	9 (53)	1.00
PDE-5 inhibitor	23 (68)	11 (65)	12 (71)	0.71
sGC	2 (6)	1 (6)	1 (6)	1.00
Diuretic	24 (71)	9 (53)	15 (88)	0.024
Anticoagulation	14 (41)	9 (53)	5 (29)	0.16
Aspirin	8 (24)	1 (6)	7 (41)	0.015
CCB	7 (21)	3 (18)	4 (24)	0.67
BB	1 (3)	0 (0)	1 (6)	0.31
Spironolactone	4 (12)	2 (12)	2 (12)	1.00

Values are presented n (%) unless otherwise indicated.

SD, standard deviation; BMI, body mass index; SBP, systolic blood pressure; DBP, diastolic blood pressure; BPM, beats per minute; PH, pulmonary hypertension; PAH, pulmonary arterial hypertension; CTD, connective tissue disease; CHD, congenital heart disease; CTEPH, chronic thromboembolic disease; WHO, World Health Organization; FC, functional class; 6MWD, 6-min walk distance; ERA, endothelin receptor antagonist; PDE-5, phosphodiesterase type 5; sGC, soluble guanylyl cyclase.

**Fig. 1.**
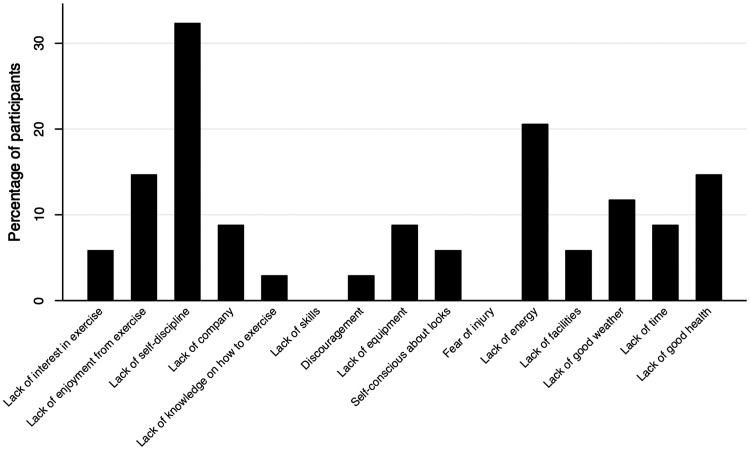
Percentage of participants reporting barrier interferes “often” or “very often.”

The top three barriers cited by participants were lack of self-discipline, lack of energy, and lack of interest in exercise (Table 2). In addition to lack of self-discipline and lack of energy, lack of enjoyment from exercise and lack of good health were the most commonly cited barriers that “often” or “very often” interfered with PA for the cohort (Fig. 1). Lack of skills and fear of injury did not “often” interfere with PA for participants in the cohort.

**Table 2.** Patient perceived barriers to PA.

Barriers (mean (SD))	Total (n = 34)	“High activity” (n = 17)	“Low activity” (n = 17)	*P* value
Lack of interest in exercise	2.5 (0.93)	2.1 (0.9)	2.9 (0.78)	0.02
Lack of enjoyment from exercise	2.4 (1)	1.9 (0.86)	3 (0.94)	0.01
Lack of self-discipline	2.8 (1.2)	2.3 (1.2)	3.3 (1.2)	0.03
Lack of company	2.2 (1.1)	1.9 (0.97)	2.4 (1.1)	0.20
Lack of knowledge on how to exercise	1.6 (0.85)	1.4 (0.71)	1.9 (0.93)	0.11
Lack of skills	1.6 (0.77)	1.4 (0.71)	1.9 (0.78)	0.08
Discouragement	2 (0.95)	1.7 (0.85)	2.3 (0.99)	0.08
Lack of equipment	1.8 (1.1)	1.5 (0.8)	2.1 (1.3)	0.17
Self-conscious about my looks when I exercise	1.6 (0.95)	1.4 (0.8)	1.8 (1.1)	>0.20
Fear of injury	1.9 (0.86)	1.8 (0.83)	1.9 (0.9)	>0.20
Lack of energy	2.6 (1.2)	2.4 (1.2)	2.8 (1.3)	>0.20
Lack of facilities	1.9 (1)	1.7 (0.92)	2.2 (1.1)	0.18
Lack of good weather	2.4 (1.1)	2.2 (0.97)	2.6 (1.3)	>0.20
Lack of time	2.2 (1.1)	2.4 (1.3)	2 (0.94)	>0.20
Lack of good health	2.4 (1.1)	2.4 (0.81)	2.4 (1.3)	>0.20
Total average barriers	2.1 (0.68)	1.9 (0.6)	2.4 (0.68)	<0.05

Mean patient perceived barriers to PA grouped by activity level. *P* value for bivariate analysis.

Perceived barriers differed by activity level among patients in the low versus high activity group (Table 2). Participants in the low activity group reported a higher average summary barrier score (*P* = 0.045) and higher average scores for several barriers including lack of interest in exercise, lack of enjoyment from exercise, and lack of self-discipline. There were trends toward more reported lack of skills and discouragement from exercise.

**Fig. 2.**
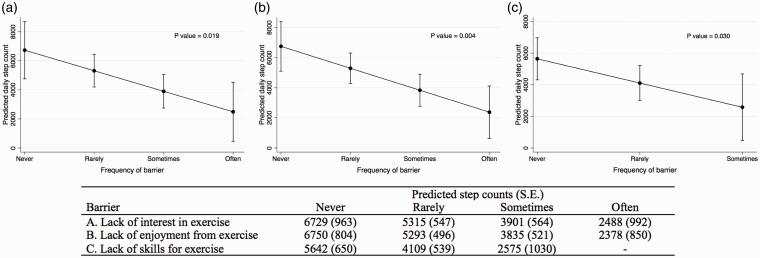
Predicted average daily steps as a result of the frequency of a barrier interfering with exercise or PA adjusted for age, sex, and etiology of PH.

After adjusting for age, sex, and etiology of PH, several barriers remained associated with lower PA (as a continuous outcome) including lack of interest in exercise, lack of enjoyment from exercise, and lack of skills for exercise (Table 3). The summary barriers score was no longer significantly associated with PA (*P* value = 0.076). For every 1-unit increase in the reported perceived lack of interest in exercise, lack of enjoyment from exercise, and lack of skills for exercise, the associated decrease in step counts was -1414 steps (95% CI = −2580 – −248), −1458 steps (95% CI = −2404 – −511), and −1533 steps (95% CI = −2910 – −156), respectively. The predicted average daily step counts in the multivariable model by the frequency of a barrier for lack of interest, lack of enjoyment, and lack of skill are shown in Fig. 2. Participants who reported “never” experiencing a lack of interest in exercise or lack of enjoyment in exercise had a predicted average daily step count of >6700 steps compared to <2500 steps among participants who “often” experienced these barriers. In the exploratory multivariable analysis, both lack of knowledge (beta coefficient = −43, 95% CI = −82 – −3.4) and lack of skills (beta coefficient = −58, 95% CI = −101 – −15) was associated with reduced 6MWD.

**Table 3.** Results of multivariable logistic regression reporting Beta coefficient for step count.

	Beta coefficient (95% CI)	*P* value
Lack of interest in exercise	−1414 (−2580 – −248)	0.019
Lack of enjoyment from exercise	−1458 (−2404 – −511)	0.004
Lack of self-discipline	−473 (−1393 – 520)	>0.20
Lack of company	−405 (−1463 – 653)	>0.20
Lack of knowledge on how to exercise	−940 (−2202 – 322)	0.138
Lack of skills	−1533 (−2910 – −156)	0.030
Discouragement	−744 (−1888 – 399)	0.193
Lack of equipment	−330 (−1395 – 736)	>0.20
Self-conscious about my looks when I exercise	−701 (−1884 – 482)	>0.20
Fear of injury	−346 (−1653 – 960)	>0.20
Lack of energy	−576 (−1558 – 405)	>0.20
Lack of facilities	−584 (−1737 – 570)	>0.20
Lack of good weather	−719 (−1654 – 216)	0.127
Lack of time	−55 (−1164 – 1054)	>0.20
Lack of good health	−474 (−1641 – 692)	>0.20
Total average barriers	−1468 (−3101 – 164)	0.076

Beta coefficient represents associated change in daily step counts per 1-unit increase in barrier adjusted for age, sex, and PAH etiology.

## Discussion

In this cross-sectional study of participants with PAH and CTEPH, we investigated the association of patient-perceived barriers on PA. Internal barriers, or barriers as a result of one’s own beliefs and decisions,^16^ were most commonly reported as interfering with PA or exercise regardless of activity level. The most commonly cited reasons included lack of self-discipline, lack of energy, and lack of interest. Higher perceived lack of enjoyment, lack of interest, and lack of skills were associated with low PA after adjusting for known predictors of PA.

The health benefits of regular daily exercise and PA are well established. PA is a known, modifiable risk factor for heart disease and has been identified as a core health behavior that contributes to cardiovascular health by the American Heart Association.^17^ Recent guidelines stress the necessity of focusing on subgroups of the population because of the unique impact of diseases on PA.^18^ The current PH guidelines recognize this and recommend encouraging PA while avoiding distressing symptoms or excessive exertion.^7,8^ Despite the proven benefits of PA, patients with PH remain sedentary.^1–3^ Participants in our study walked far fewer steps compared to most adults in the United States.^19^ Importantly, lower daily PA in patients with PH has been shown to be associated with worse patient-reported symptoms^1^ and interventions targeting PA are being considered as a therapeutic goal in the treatment of PH.^20^

The best way to optimize PA in patients with PH is unclear, though a combination of targeted pharmacological therapies, structured exercise, and application of behavioral change theory are likely to result in increases.^9^ Determinants of PA are complex and differ between populations of patients.^21–23^ PA levels are an interplay of not only the individual and their medical conditions but are also impacted by social, environmental, and policy contributions.^23^ Importantly, the identification of barriers has been found to be associated with an increased impact of interventions aimed at increasing PA.^24^ As a result, understanding the correlations of patient-perceived barriers and PA is a necessary first step toward optimizing PA.

In our study of participants with PH, the individual or internal barriers, rather than external barriers, correlated with PA levels. Broadly examining the inherent satisfaction participants obtain from PA,^25^ these internal patient factors have been shown to impact motivation for exercise in diverse populations with chronic cardiac disease.^26^ Strategies seeking to encourage increased PA need to acknowledge this and consider interventions that increase intrinsic motivation and promote participant enjoyment.^27^ One such strategy to improve PA that has been shown to be effective is to focus on these intrinsic motivations rather than just emphasizing the health benefits of PA.^28^ The optimal scenario will include individualized tailoring of the PA plan and/or interventions in trials focused on skill improvement, personal accomplishment, and excitement while performing PA.^25^ Additionally, in the exploratory analysis, similar barriers were associated with decreased sub-maximal exercise capacity (6MWD) suggesting that strategies that increase PA may be associated with changes in this well-established outcome.

Interestingly, total average barriers and external barriers including environmental factors such as lack of equipment, poor weather, and lack of time did not correlate with reduced PA in our study. While total average barriers showed a trend toward an association, this was not statistically significant. We suspect our study did not have adequate power to identify a difference though this will need to be confirmed in future studies. With regard to external barriers, prior work in different patient populations has shown a large impact of the social and physical environment on PA.^23^ There are several possible explanations for this finding. It is recognized that population subgroups experience different barriers that impact PA.^18^ Older adults have previously been shown to be impacted more by environmental variables and the relatively younger age of patients with PH may explain the lack of an association between environmental barriers and PA.^29^ Additionally, how PA is measured could influence the results. A strength of the study that differentiates it from much of the prior work was the use of activity trackers to objectively measure activity.^23^ It is possible that if patient-reported PA were used, the associations would change. Regardless, confirming the lack of an impact of external barriers on PA in patients with PH with longitudinal data has important trial design and policy implications.

There are limitations of our study worth discussing. Our participants were enrolled at a single, academic center and may not be representative of the general PH population. The comparatively large sample size for a study of ambulatory PA monitoring does alleviate this concern to a degree.^1–3^ This was a cross-sectional analysis that is unable to address causality. However, much of the initial work on barriers to PA in other disciplines has been cross-sectional^23^ as it lays the foundation for future work. Being an observational study, the impact of confounding must always be considered. We did adjust for PAH etiology, an established risk factor for low PA.^5^ We were unable to adjust for additional potential confounders because of sample size limitations. As mentioned, though the sample size is small, it is comparable to studies that have objectively measured PA.^1–3^ Lastly, 15% of participants did not have valid step counts for the two-week period. While this is similar to the recently completed Electronic Activity Level Monitoring Pilot in Pulmonary Hypertension study,^30^ a better understanding of barriers to PA monitoring among patients with PH is necessary. A recently completed home exercise pilot study suggests that scheduled verbal communication may improve adherence in future studies.^31^

These findings have important implications in the understanding of PA levels in patients with PH and the design of future intervention trials. For a clinician currently caring for patients with PH, these findings emphasize the need to understand a patient’s internal barriers and design individual PA plans to make PA a habit.^32^ Tailoring an exercise plan could have a profound impact on PA if addressing barriers increases activity as previously shown.^24^ For example, if a patient reported not enjoying PA, one could counsel the patient on finding an activity they enjoy such as going for a bike ride with friends. Given the associated increase in PA by almost 1500 steps when a barrier was reduced (Fig. 2), this would be well above the minimal important difference of 600–1100 steps described in studies of pulmonary rehabilitation for chronic lung disease.^33^ In regards to trial design, future interventional studies aimed at increasing PA should recognize that a one-size-fits-all approach may not be the most efficacious. While increasing access to proven therapies such as pulmonary rehabilitation programs will improve PA, future work aimed at optimizing PA in patients with PH should include multidimensional interventions that consider the potential impact of intrinsic barriers. Including patients in the design of such work through qualitative or mixed-methods research has the potential to maximize motivation.^34^ In the meantime, identifying and addressing the intrinsic barriers with an individualized plan has significant potential while waiting for completion of studies aimed at increasing PA.

In conclusion, several barriers including lack of self-discipline, lack of energy, and lack of interest are the most commonly cited reasons preventing increased exercise and PA. Barriers related to enjoyment, interest, and skills were associated with PA levels. Personalized regimens that address these barriers have the opportunity to increase PA though additional research is needed to determine causality.
